# Items

**Published:** 1884-03

**Authors:** 


### Items.

THE following circular letter has been distributed in Chicago, and deserves the attention of all our readers, as its object is in the highest degree commendable :

No. 6 SIXTEENTH ST, CHICAGO, ILL, Dec. 5, 1883.

*Dear Doctor :*

The Trustees of the Public Library, after conference with the Chicago Medical Society, have resolved to found a Medical Department in the Library, and to make appropriations for its maintenance and increase. As the public money available for this purpose is limited, the Chicago Medical Society has voted to expend $500 of its surplus funds to aid the enterprise, and has directed its Library Committee to appeal to every physician, dentist, and pharmacist to give a small sum for the same purpose. There are over 1,500 members of these three professions in the county, and a donation of two dollars from each will purchase a large number of practical works on Medicine, Dentistry, and Pharmacy, such as every one of them needs to consult. The Public Library will preserve the works, add to their number, and insure them against fire. They will not loan them out, but keep them always ready to be consulted and read in the Library rooms. We ask you, therefore, to kindly coöperate with us by sending two dollars in the enclosed envelope, with your name and address, to Dr. Edmund Andrews, the Chairman of the Committee on Library, to be expended for this object.

Very respectfully yours,

EDMUND ANDREWS, M.D., Chairman,
No. 6 Sixteenth St.
OSCAR DEWOLF, M.D.,
F. C. HOTZ, M.D.
Library Committee of the Chicago Medical Society.

COATED TONGUES.

The microscope gives the following as some of the articles found by microscopical examination on coated tongues: Fibers of wood, linen and cotton; fibers of spiral vessels; fibers of muscle, in one case eight hours after eating; starch grains; cheese mold; portions of potato skin; scales; moths, etc.; hairs from legs of bees; hairs from legs of spiders; pollen of various flowers; stamens of various flowers; hairs of cats, quite common; hairs of mouse, once only; hairs from various leaves; wing of mosquito once; fragments of the leaves of tobacco; chamomile flowers, etc. A list ten times as long could be made, but *jam satis.*

DECALCIFIED BONE DRAINAGE TUBES.—Professor Gross gives the following directions for making bone-drainage tubes. Procure the femur and tibiæ of a chicken or turkey, take off the periosteum, and place the bones in 16⅔ per cent. solution of official hydrochloric acid until they become soft; then cut off the ends and foree out the endosteum; replace in the hydrochloric acid solution until they become very soft; fill them with horsehairs, which must be removed if pus forms, as they will not allow it to pass. However, he recommends removing the bone tube in twenty-four hours, as it can only be absorbed by granulations, which render union by first intention out of the questton.—*College and Clinical Record.*

AT the recent annual meeting of the Fox River Medical Association, held at Geneva, Dr. Catharine B. Slater, of Aurora, was elected President. This Association is composed of the physicians of all towns in the Fox River Valley, and for a number of years had been presided over by Dr. Tefft, of Elgin. Dr. Slater will fill the position with dignity and ability, and with credit to herself and the association, though we believe there has been but one other instance in the West where a lady physician has been similarly honored. In the Aurora Medical Society also her talents seem to be duly appreciated, since she is officiating as secretary and treasurer of that organization.

THE New England railroads are credited with having killed in the past year 221 persons, and with having injured 535.

